# Effect of Pulse Frequency on Microstructure and Mechanical Properties of 2198 Al-Li Alloy Joints Obtained by Ultrahigh-Frequency Pulse AC CMT Welding

**DOI:** 10.3390/ma12010079

**Published:** 2018-12-26

**Authors:** Liwei Wang, Yingchao Suo, Chaofeng Wu, Dianlong Wang, Zhimin Liang

**Affiliations:** School of Materials Science and Engineering, Hebei University of Science and Technology, Shijiazhuang 050018, China; wangliwei110127@163.com (L.W.); suoyingchao@163.com (Y.S.); chaofengwu2018@163.com (C.W.); lianghebust@163.com (Z.L.)

**Keywords:** AC CMT, ultrahigh-frequency pulse current, 2198 Al-Li alloy, microstructure, mechanical properties

## Abstract

In this study, 2198 Al-Li alloy, a low density and high-performance material for aerospace equipment, was welded using ultrahigh-frequency pulse alternating current with cold metal transfer (UHF-ACCMT). Influence of different ultrahigh-frequency on the formation, porosity, microstructure, microhardness and tensile strength of the welded joints were investigated. The results showed that the coupled ultrahigh-frequency current generated electromagnetic force to stir the liquid metal of molten pool. The weld formation became much better with metallic luster and uniform ripples at frequency of 60 kHz and 70 kHz. The porosity was the minimum at frequency of 60 kHz. Furthermore, the molten pool was scoured and stirred by the electromagnetic force which provided the thermal and dynamic conditions for nucleation and grain refinement, the width of fine equiaxed grain zone became larger, and the number of equiaxed non-dendrite grains increased. With the grain refining and crystallize transition, the average microhardness and tensile strength of the joints at frequency of 60 kHz reached up the highest value, 116 HV0.1 and 338 MPa, respectively. The fracture of the welded joints presented the characteristics of quasi-cleavage fracture.

## 1. Introduction

Al-Li alloys are good structural materials for aerospace due to low density, high strength, excellent performance at low temperatures, and high resistance for stress corrosion. Compared with the second generation of Al-Li alloys, the third generation of Al-Li alloys have reduced content of Li element whereas they have increased Cu, Zr, Mn and Zn elements, leading to enhanced strength and hardness, and lower anisotropy [1,2]. However, Al-Li alloys are difficult to be welded because of hot cracking and porosity formed during the welding process which can be improved through careful preparation for surface, use of suitable filler materials, and optimization of welding parameters. Therefore, choosing suitable welding parameters is critical to improving quality and properties of Al-Li welded joints.

The popular methods of welding Al-Li alloys include arc welding, laser welding, friction stir welding, etc. [3,4,5]. Compared with conventional arc welding, laser welding has lower heat input that can reduce quantity of the pores and cracks during welding. Friction stir welding (FSW) has advantages of decreasing radiation, sparking, weld cracks, porosity and shrinkage defects. Furthermore, it does not need to groove for middle thick plates welding. FSW is thus often used in the welding of high strength aluminum alloy for aircraft. However, laser welding of Al-Li alloys has some limitations such as high reflection, intense vaporization due to low boiling point of alloying elements, and wide focal spot during laser welding. FSW can only be applied to a relatively simple, long and straight weld seam, and presence of the key hole at the end of seam is a great defect [6,7,8,9].

Lowering heat input and cleaning oxide-film are effective ways to minimize the formation of pores in the weld metal of Al-Li alloys [10,11]. Direct current (DC) CMT welding of aluminum alloys has therefore been used [12,13]. Reduction of heat input and generation of metal compounds in welding process can reduce cracking of weld [14]. It was also found that solidification structure of the weld pool cannot be controlled under conditions of low heat input and free growth of crystal grains [15,16]. Combined structures of coarse equiaxed dendrites and fine equiaxed non-dendrite resulted in strength loss of the welded joints. Using the advantage of cleaning oxide-film, AC CMT with positive polarity pulse (EP-CMT-phase) and negative polarity pulse (EN-CMT-phase) has been proposed to effectively control the crystallization process of the weld pool, refine grains, and further improve properties of the joints [17,18].

Frequency of pulse current can significantly refine solidified microstructure of aluminum and titanium alloy [19,20]. Within a certain range, the higher the frequency of pulse current is, the smaller the grains are. In alternating polarity arc welding coupled with high-frequency pulse current, reasonable frequency of pulse current is likely to strongly affect solidification structure and crystallization process of the weld pool, which will finally improve mechanical properties of the welded joints [21,22,23,24]. The AC CMT coupled with ultrahigh frequency current between 20 kHz and 80 kHz was first adopted to weld 2198 Al-Li alloy in this paper. Combining test measurements and welding metallurgy, the effects of pulse current frequency on the formation, porosity, microstructure, microhardness and tensile strength of the welded joints were studied. Under the action of ultrahigh-frequency pulse current, the interaction mechanism of microstructure and mechanical properties of the welded joints was studied.

## 2. Experimental Procedures

**Figure 1:**
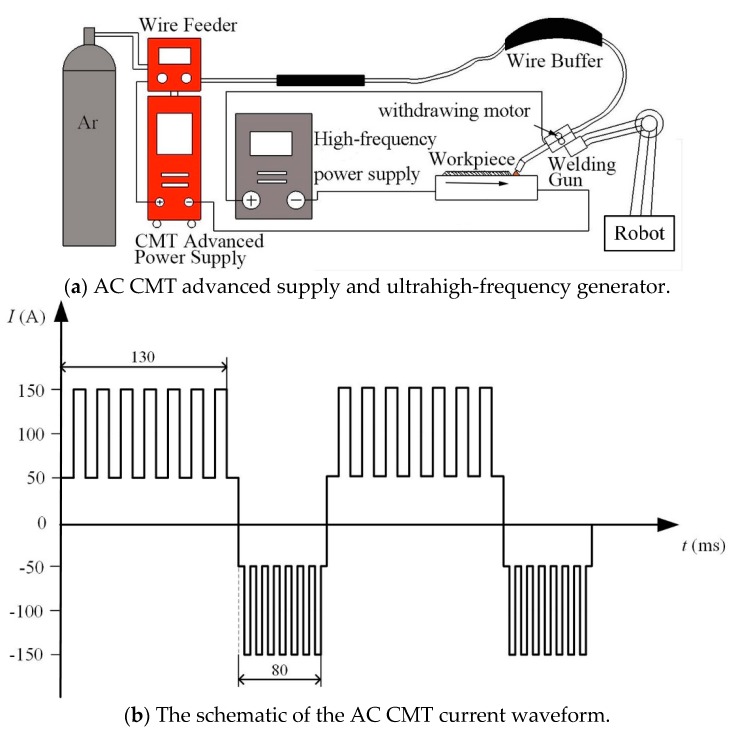
The experiment installation of AC CMT coupled with ultrahigh-frequency power supply (**a**) and the schematic of the AC CMT current waveform (**b**).

Compared to the conventional welding methods, the AC CMT mode of Fronius CMT Advanced 4000 machine has welding currents in electrode positive (EP) phase and electrode negative (EN) phase. There are significant differences between EP and EN phases. In the EP phase, Ar ions hit the molten pool and surface of the work piece, which can quickly clean oxide film on the surface of the workpieces. In the EN phase, the arc is relatively constricted and more heat is redirected to the wire, resulting in fast melting of the wire. Compared with EN phase, arc in EP phase is more divergent and higher heat is imposed on the workpieces, which will increase heat-input and improve quality of the welded joints [25,26,27]. In this study, the welding current in EP phase was therefore selected to couple with pulse current of ultrahigh-frequency to improve the arc characteristics. Two relatively independent power systems were connected in a parallel way, and then linked to the same welding gun held by the KUKA KR60 robot, as shown in Figure 1a. The current waveform of AC CMT is shown in Figure 1b, the average current *I* of AC CMT is 100 A. The base current *I_b_* of ultrahigh-frequency pulse alternating current with cold metal transfer (UHF-ACCMT, UHF100, HEBUST, Shijiazhuang, China) is 85 A, the peak current *I_p_* of UHF-ACCMT is 30 A, duty cycle *δ* is 50%, and frequency are 20 kHz, 30 kHz, 40 kHz, 50 kHz, 60 kHz, 70 kHz and 80 kHz, respectively. Therefore, the total current of UHF-ACCMT *I_t_* = *I_b_* + *I_p_* × *δ* = *I*. The specific parameters are listed in Table 1.

2198-T8 Al-Li alloy plates in size of 135 mm × 95 mm × 2 mm and aluminum-silicon (Al-Si) wire ER4043 with a diameter of 1.2 mm were used as the base material and the additional welding wire. The ER4043 wire can provide a large number of Al-Si eutectics during welding [28]. Eutectics have good filling capacity of reducing hot cracks [29]. In order to eliminate macropores effectively, oxide film on both sides of the butt with square welding groove and without root gap (at least 0.15 mm thickness) was cleaned using wire brush and scraper, and grease as well as water stains were erased with acetone [30]. Chemical composition of 2198 Al-Li alloy and Al-Si wire ER4043 are listed in Table 2. Flat welding, using guide arc plate and crater arc plate and shielding gas of pure argon, was adopted.

**Figure 2:**
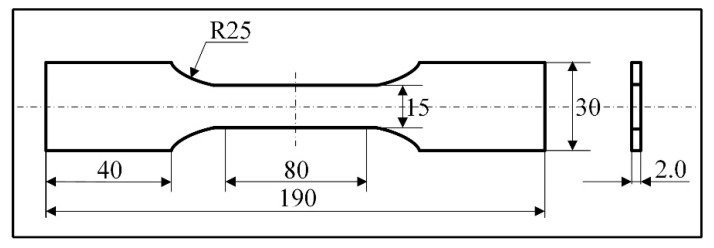
Schematic of the tensile sample (mm).

The optical microstructure of the welded joints was investigated using German Zeiss microscope of Axiovert.A1. The samples were grinded, polished and then etched with Keller reagent (2.5% HNO_3_ + 1.5% HCL +1.0% HF +95% H_2_O, volume percentage). Vickers microhardness test was carried out with a 100 g load and 15 s dwell time. Data were acquired with a distance of 0.5 mm across the mid-depth longitudinal section of the welded joints. Tensile tests were performed under the standard of ISO 4136: 2001 on SANS CMT5204 electronic universal testing machine (Shenzhen, China) at a speed of 1 mm/min. Dimension of the tensile samples is shown in Figure 2.

## 3. Results and Discussion

### 3.1. Macroscopic Morphology of the Welded Joints

**Figure 3:**
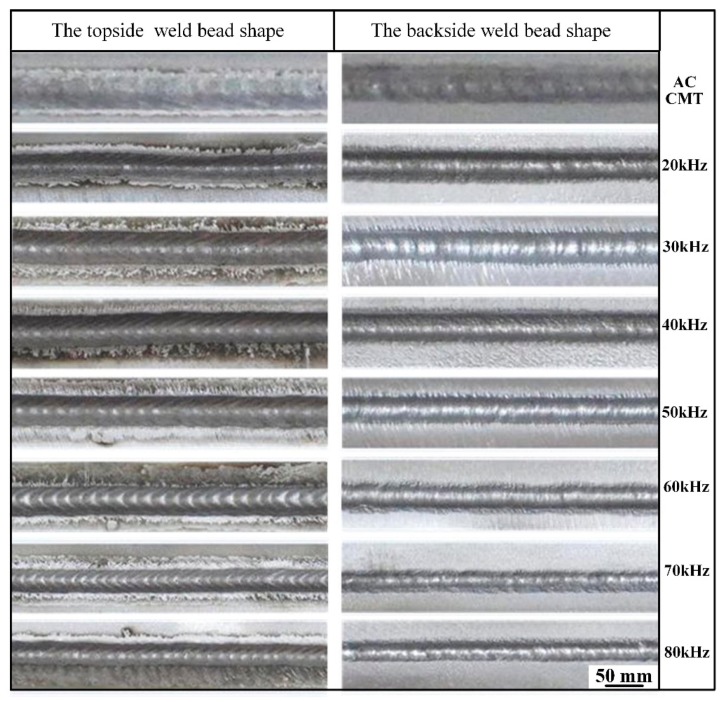
The topside and backside weld bead shape welded by AC CMT and AC CMT coupled current at frequency of 20 kHz, 30 kHz, 40 kHz, 50 kHz, 60 kHz, 70 kHz, and 80 kHz, respectively.

Figure 3 shows the welds appearance of 2198 Al-Li alloy welded by AC CMT coupled with ultrahigh-frequency pulse current. Compared with AC CMT process, the base materials exhibit less deformation and surface of the joints are smooth and homogeneous by AC CMT coupled with ultrahigh-frequency pulse current. Metallic luster and uniform ripples can be seen on the welds surface. There are no concave, undercut, weld beading, incomplete penetration, slag or other visual defects. However, the welds appearance have obviously changed with varying frequency, ripples are more obvious and regular by coupling with ultrahigh-frequency current of 60 kHz and 70 kHz, and it almost disappeared at a frequency of 20 kHz. With other ultrahigh-frequency current, ripples have been weakened by different degrees.

According to the electromagnetic theory, the coupled ultrahigh-frequency current generated arc force, which plays a part in stirring the liquid metal in molten pool [31]. The flow form and flow rate of liquid metal are determined under the combined action of surface tension and electromagnetic force, so the solidification morphology of molten pool is different with varying pulse current at different frequency.

### 3.2. Pores in Welded Joints

**Figure 4:**
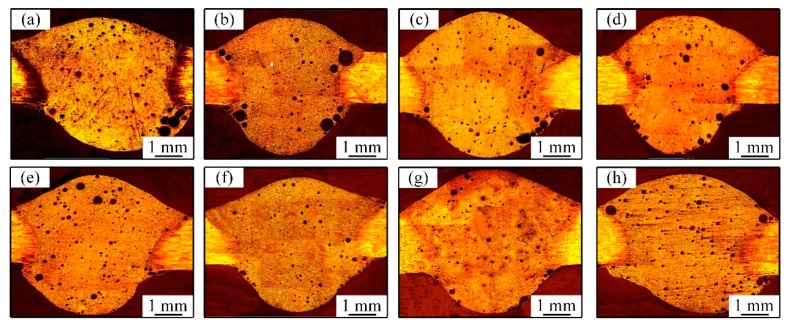
(**a**–**h**) Distribution of pores in the welded joints welded by (**a**) AC CMT and (**b**) AC CMT coupled current at frequency of 20 kHz, (**c**) 30 kHz, (**d**) 40 kHz, (**e**) 50 kHz, (**f**) 60 kHz, (**g**) 70 kHz, and (**h**) 80 kHz, respectively.

Figure 4 shows the distribution of pores in welded joints. The pores were mainly hydrogen hole, which was difficult to eliminate during welding of Al-Li alloys [31,32,33,34]. It is known that the production of welding pores was attributed to the fact that hydrogen in liquid droplet cannot grow up and overflow due to the low heat input and quick solidification of molten pool during CMT welding. On the one hand, the purity of argon, the cleaning degree of work piece before welding and the high environment humidity were the possible reason of excessive porosity in this study. Figure 4a shows the pores distribution in welded joints welded by AC CMT, in which many pores have relatively large size and are distributed randomly in the welded joints. Figure 4b–d show the pores’ distribution in welded joints welded by AC CMT coupled current at frequency of 20 kHz, 30 kHz, and 40 kHz, respectively. The total number as well as the size of pores presented a decreasing trend. The bigger pores are mainly distributed in the edge of the welded joints, the smaller pores were seen mainly in the middle of the welded joints. Figure 4e–h show the pore distribution in welded joints welded by AC CMT coupled current at frequency of 50 kHz, 60 kHz, 70 kHz, and 80 kHz, respectively. It is seen that the number of pores became smaller and the size of pores became much smaller. A small number of little ones existed in the middle of the welded joints, the bigger pores existed in the edge of the welded joints. A small number fraction of pores is shown in Figure 4f with the coupled ultrahigh-frequency pulse current at a frequency of 60 kHz. According to the standard of ISO-10042:2005, the porosity of the AC CMT joint is 3%, which is D grade (Figure 4a). The joint welded by AC CMT coupled current at frequency of 60 kHz has only a porosity of 1%, which is B grade (Figure 4f). Compared with AC CMT, the porosity and pore diameter of UHF-ACCMT were significantly reduced.

**Figure 5:**
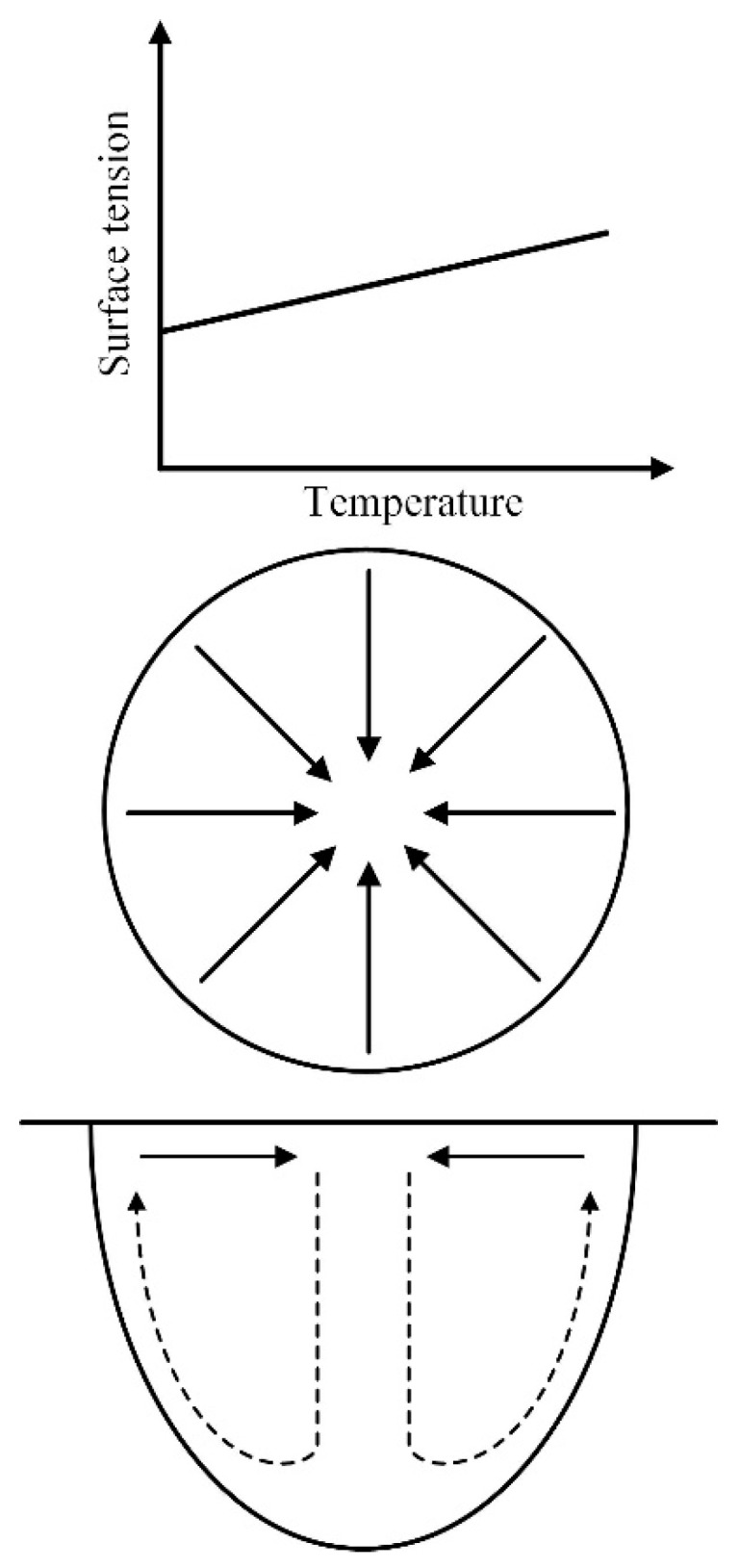
Relation between surface tension distribution and liquid metal flow of the molten pool.

The coupled ultrahigh-frequency pulse current generated electromagnetic force that played an important role in stirring the liquid metal in molten pool. Meanwhile, Si is a strong surface active element in ER4043 that makes the surface tension of liquid metal directly proportional to temperature gradient [35]. The liquid metal was driven by electromagnetic force and surface tension, then it flowed down along the center of molten pool and up along the edge of molten pool, as shown in Figure 5. It benefited the bubbles escaping and reducing the tendency of weld porosity when the direction of fluid flow was consistent with the rise of bubbles. The number fraction of porosity was the minimum when the frequency was 60 kHz.

### 3.3. Microscopic Structure of the Welded Joints

#### 3.3.1. The Microscopic Structure of the Fine Equiaxed Grain Zone (EQZ)

**Figure 6:**
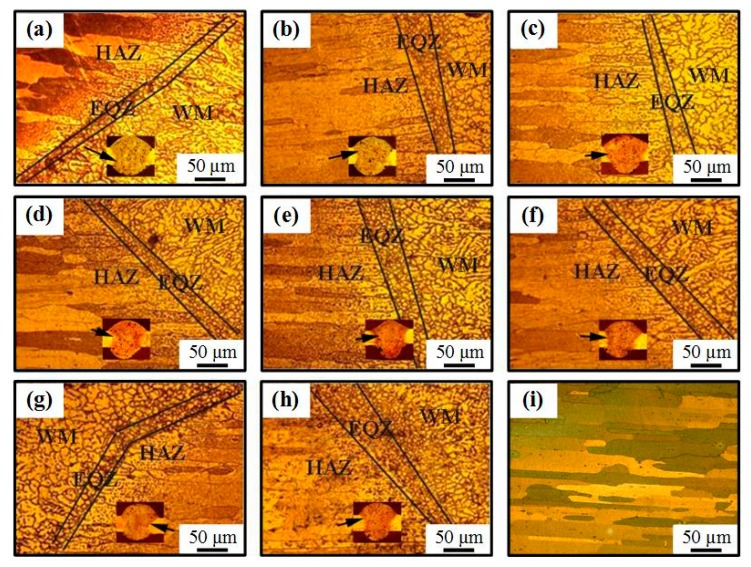
(**a**–**i**) The optical microstructure of the welded joints welded by (**a**) AC CMT and (**b**) AC CMT coupled current at frequency of 20 kHz, (**c**) 30 kHz, (**d**) 40 kHz, (**e**) 50 kHz, (**f**) 60 kHz, (**g**) 70 kHz, (**h**) 80 kHz and BM (**i**), respectively.

Figure 6 shows the optical microstructure of the welded joints and base metal (BM). Compared to the heat affected zone (HAZ) and weld metal (WM), the EQZ welded by AC CMT is composed of fine equiaxed grains, as shown in Figure 6a. Lin et al. [36] found that the region was not only recrystallized, but also the region was affected by the tiny particles in the base material, such as Al_3_Ti and Al_3_Zr, which provided a large number of heterogeneous nucleation sites during the solidification that promoted the formation of heterogeneous nucleation in the EQZ [36].

The width of fine EQZ became bigger when coupled with the ultrahigh-frequency pulse current, as seen from Figure 6b–h. The coupled ultrahigh-frequency current flowed through molten pool. A high frequency pulsed electromagnetic field was generated inside molten pool. Under the high frequency pulsed electromagnetic force, the liquid metal presented periodic vortex motion which scoured the boundary of fusion zone and heat affected zone. Some unfused high melting point particles in the boundary, such as Al_3_Ti and Al_3_Zr, appeared in the molten pool and they became the heterogeneous nucleation sites which formed the wider fine EQZ [37,38,39]. The microscopic structure of BM presents lath-shaped along the rolling direction, as shown in Figure 6i.

#### 3.3.2. The Microscopic Structure of the WM

**Figure 7:**
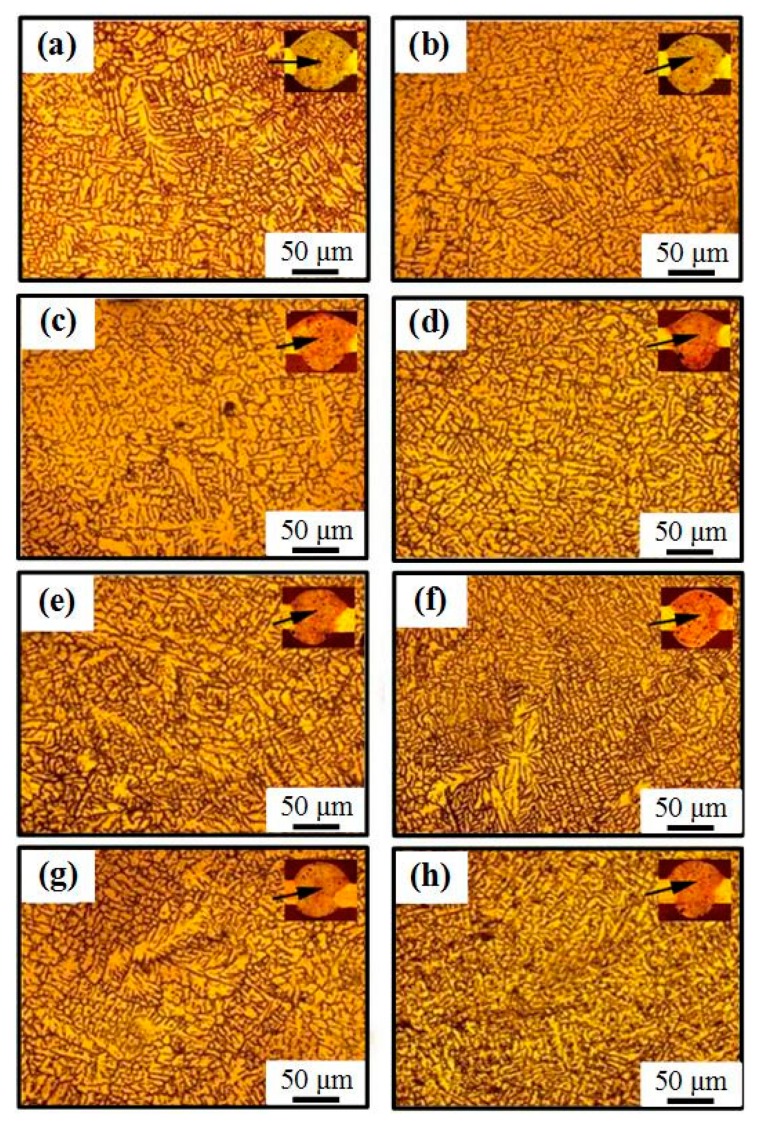
(**a**–**h**) The optical microstructure of WM welded by (**a**) AC CMT and (**b**) AC CMT coupled current at frequency of 20 kHz, (**c**) 30 kHz, (**d**) 40 kHz, (**h**) 50 kHz, (**e**) 60 kHz, (**f**) 70 kHz, and (**h**) 80 kHz, respectively.

Figure 7 shows the optical microstructure of WM. It was coarse dendrite grains in the WM of the welded joints welded by AC CMT, as shown in Figure 7a. When 2198 Al-Li alloy was welded by AC CMT coupled ultrahigh-frequency current, grain refinement occurred obviously in the WM, as shown in Figure 7b–h. The equiaxed dendrite grains appeared in the WM at a frequency of 20 kHz in Figure 7b. When the frequency of pulse current is 30 kHz and 40 kHz, the fine equiaxed non-dendrite grains appeared in the WM, alternating with the distribution of equiaxed dendrite grains in the middle of the weld, as shown in Figure 7c,d. Micrographs of the WMs in Figure 7e–g show that a large amount of fine equiaxed non-dendrite grains are non-uniformly distributed in the WM at frequency of 50 kHz, 60 kHz and 70 kHz, respectively. However, when the frequency exceeds 70 kHz, grain refinement began to weaken, the number fraction of the equiaxed non-dendrite structures reduced, and there are mainly equiaxed dendrites in WM (Figure 7h).

The electromagnetic force generated by the coupled ultrahigh-frequency current played a major role in stirring the liquid metal in molten pool, which can effectively break new grains and provide the sites of heterogeneous nucleation [23]. On the other hand, some high melting point particles, such as Al_3_Ti and Al_3_Zr, were involved into molten pool under the action of liquid metal scouring, which provided the sites of the heterogeneous nucleation [37,38,39]. In addition, according to the principle of metal crystallization thermodynamics, the regular flow of liquid metal in molten pool was conducive to promoting the homogeneity of liquid metal temperature, which can effectively reduce the temperature gradient at the front of the solid-liquid interface and enhance component undercooling [40]. The increase of undercooling promoted the microstructure transformation of WM from coarse dendrite grains to fine equiaxed grains, which significantly played a role in grain refinement.

### 3.4. Mechanical Properties of the Welded Joints

#### 3.4.1. Microhardness of the Welded Joints

**Figure 8:**
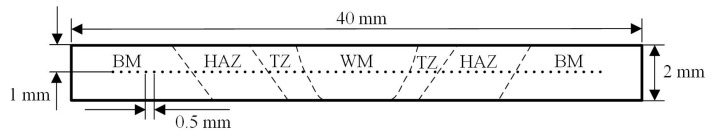
Schematic drawing of microhardness measuring points.

**Figure 9:**
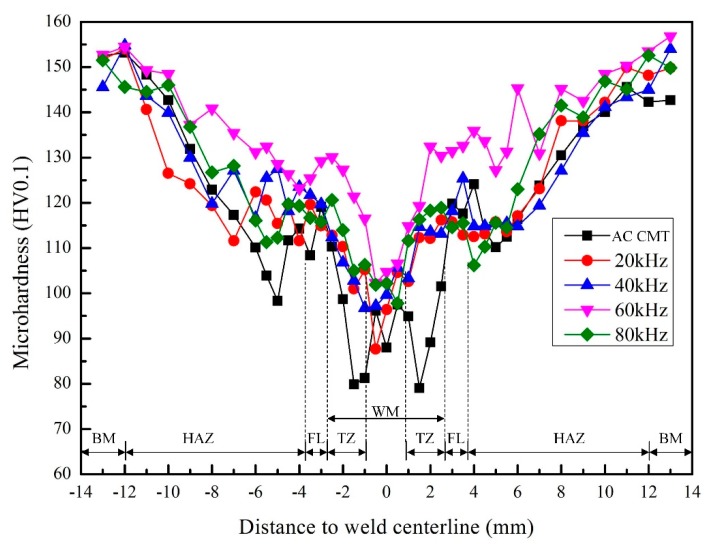
Microhardness distribution of the welded joints welded by AC CMT and AC CMT coupled current at frequency of 20 kHz, 40 kHz, 60 kHz, and 80 kHz, respectively.

Three samples of different processes were prepared for microhardness testing. The microhardness measuring points are shown in Figure 8. Figure 9 shows the averaged microhardness distribution of the welded joints. The microhardness curves had the similar variation tendency with different frequency of pulse current. The microhardness of base material (BM) was the highest, about 150 HV0.1. The microhardness decreases gradually from the base material (BM) to the fusion line (FL). The microhardness of weld center was higher than that of FL. The microhardness of the transition zone (TZ) between the weld metal (WM) and the FL was the lowest which became the weakest zone of the welded joints. When coupled with ultrahigh-frequency pulse currents, the microhardness in different regions was higher than that of uncoupled ultrahigh-frequency pulse current. Such as, the microhardness in weld center increased from 88 HV0.1 to 104 HV0.1. When the frequency of coupled pulse current was 60 kHz, the average microhardness of WM was the highest, 116 HV0.1. The increase of microhardness was related to the grain refinement, which was obvious when the coupled frequency was 60 kHz, especially, the finer grains correspond to the highest microhardness of the WM [41,42]. The transition zone between the WM and the FL is the softening zone where the metal experienced overaging caused by the thermal cycle and the strengthening phase particles agglomerated. Therefore, the coherent relationship between precipitated phase and parent phase was destroyed, resulting in an increase in size and a decrease in the number of precipitated strengthening phases within the grains, which decreases the microhardness of softening zone [43].

#### 3.4.2. Tensile Properties of the Welded Joints

**Figure 10:**
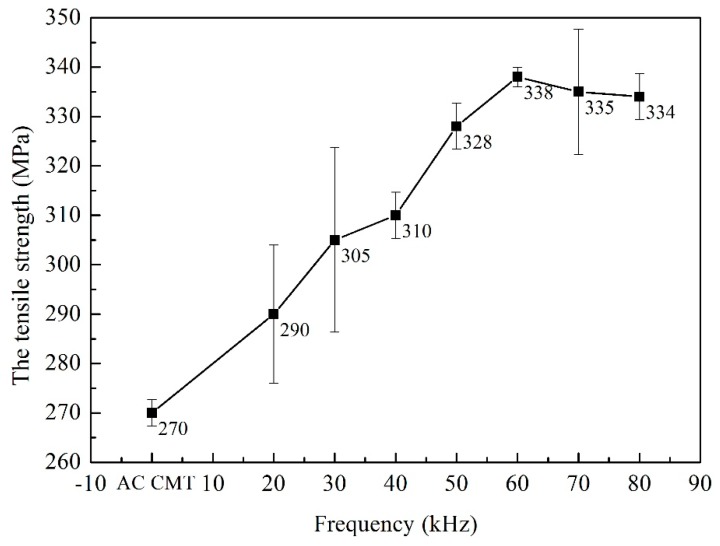
The tensile strength of joints welded by AC CMT and AC CMT coupled current at frequency of 20 kHz, 30 kHz, 40 kHz, 50 kHz, 60 kHz, 70 kHz and 80 kHz, respectively.

**Figure 11:**
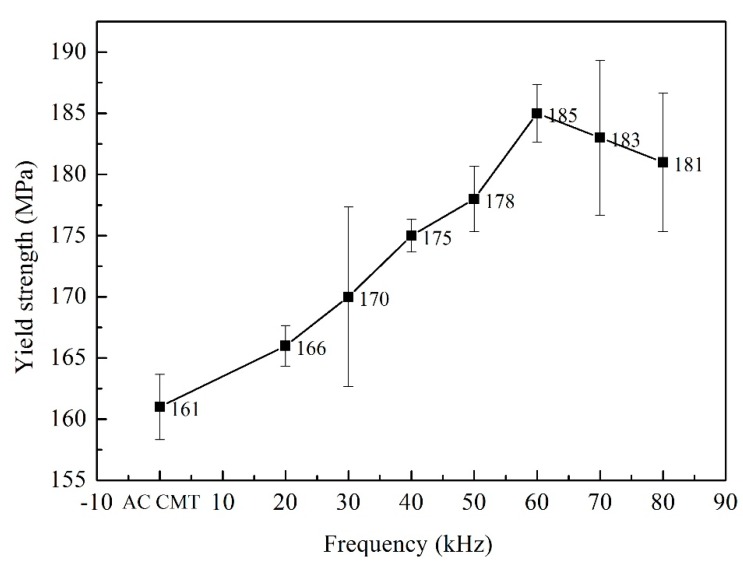
The yield strength of joints welded by AC CMT and AC CMT coupled current at frequency of 20 kHz, 30 kHz, 40 kHz, 50 kHz, 60 kHz, 70 kHz and 80 kHz, respectively.

**Figure 12:**
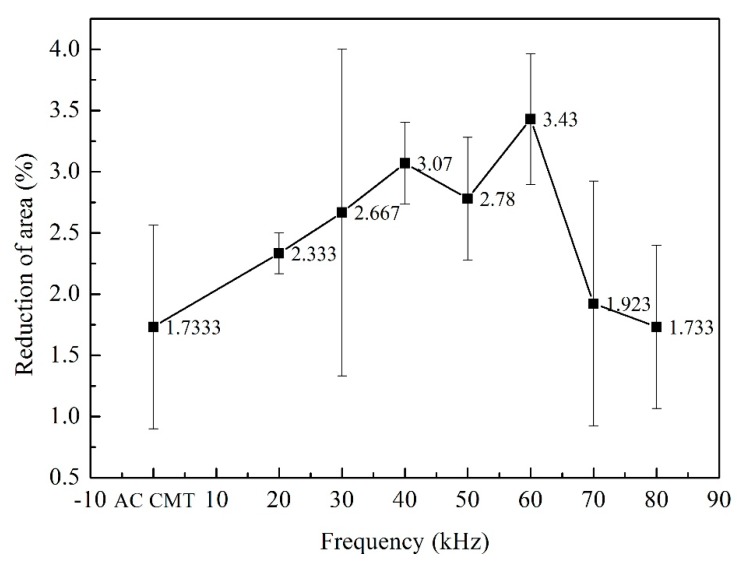
The reduction of area of joints welded by AC CMT and AC CMT coupled current at frequency of 20 kHz, 30 kHz, 40 kHz, 50 kHz, 60 kHz, 70 kHz and 80 kHz, respectively.

**Figure 13:**
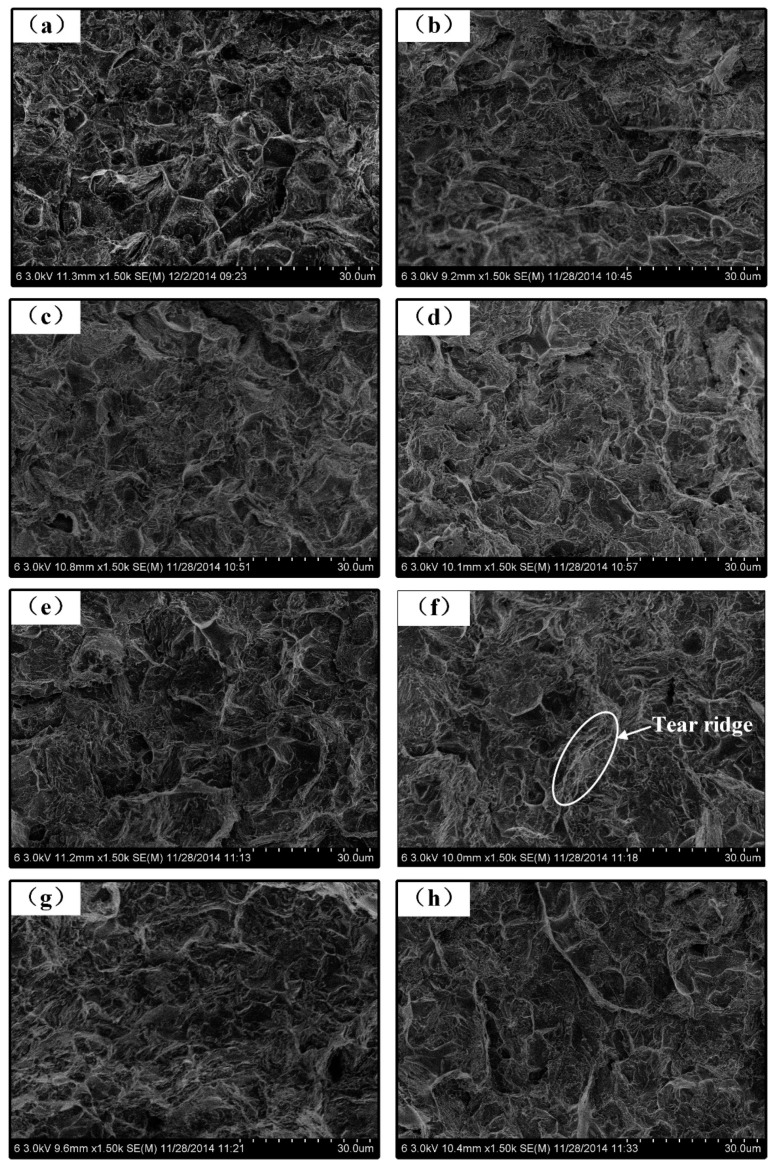
(**a**–**h**) SEM fracture surface welded by (**a**) AC CMT and (**b**) AC CMT coupled with the frequency of 20 kHz, (**c**) 30 kHz, (**d**) 40 kHz, (**e**) 50 kHz, (**f**) 60 kHz, (**g**) 70 kHz, and (**h**) 80 kHz, respectively.

Three tensile test samples of different processes were prepared. The fractures occurred at the fusion line. The tensile strength, yield strength and reduction of area of the welded joints were shown in Figure 10, Figure 11 and Figure 12. As shown in Figure 10, the tensile strength of the welded joints uncoupled with ultrahigh-frequency pulse current was 270 MPa which was only 57.4% of the base material (BM, 470 MPa). The fracture presented the characteristics of intergranular fracture, as shown in Figure 13a.The tensile strength of the welded joints increased significantly with the increasing of pulse frequency (Figure 10). When the frequency was 60 kHz, because of the wider fine EQZ and finer equiaxed non-dendrite grains in the WM, tensile strength of the joint reached up the highest value, 338 MPa. As shown in Figure 13f, a large amount of tear ridges was observed at the fractured surface and the fractured mode is the quasi-cleavage fracture. The cavities inside grains and impurities are the quasi cleavage crack source. The change of yield strength and reduction of area were similar to the tensile strength as shown in Figure 11 and Figure 12.

## 4. Conclusions

In this study, 2198 Al-Li alloy was welded using UHF-ACCMT. Influence of different ultrahigh-frequency on the formation, porosity, microstructure, microhardness and tensile strength of the welded joints were investigated. Conclusions are drawn as follows:

(1) According to the electromagnetic theory, the coupled ultrahigh-frequency current generated electromagnetic force which played a part in stirring the liquid metal in molten pool. The welded joints were smooth and homogeneous; metallic luster and uniform ripples can be seen on the welds surface when the frequency of coupled pulse current were 60 kHz and 70 kHz.
(2) Under the action of electromagnetic force and surface tension, the flowing liquid metal was conducive to bubbles escaping. The size and number of the weld pores decreased with the increase of coupled current frequency. The porosity was the minimum when the frequency was 60 kHz.
(3) The molten pool was scoured and stirred by the electromagnetic force which provided the sites of heterogeneous nucleation for the nucleation and grain refinement. The width of fine EQZ became larger, and a large amount of equiaxed non-dendrite grains was observed in the WM at frequency of 50 kHz, 60 kHz, and 70 kHz, respectively.
(4) When the frequency of the coupled pulse current was 60 kHz, the weld porosity was the minimum and grain size in WM were the smallest. The average microhardness of WM and tensile strength of the welded joints were the highest, 116 HV0.1 and 338 MPa, respectively. The fracture mode of the welded joints is quasi-cleavage fracture.

## Figures and Tables

**Table 1:** Process parameters of UHF-ACCMT welding.

Base Current *I_b_*/A	Pulse Current *I_p_*/A	Welding Rate *v*/(cm·min^−1^)	Gas Flow Rate *q*/(L·min^−1^)	EP Duration (ms)	EN Duration (ms)
85	30	70	20	130	80

**Table 2:** Composition of the base materials and wire (wt %).

Materials	Cu	Li	Zn	Mn	Mg	Si	Fe	Al
2198	2.9–3.5	0.8–1.1	≤0.35	≤0.5	0.25–0.8	≤0.08	≤0.01	Bal.
ER4043	0.3	-	0.1	0.05	0.05	4.5–6.0	0.8	Bal.
